# A new hybrid algorithm for three-stage gene selection based on whale optimization

**DOI:** 10.1038/s41598-023-30862-y

**Published:** 2023-03-07

**Authors:** Junjian Liu, Chiwen Qu, Lupeng Zhang, Yifan Tang, Jinlong Li, Huicong Feng, Xiaomin Zeng, Xiaoning Peng

**Affiliations:** 1 Department of Statistics, College of Mathematics and Computer Science, Hunan Normal University, Changsha, 410081 Hunan China; 2 Department of Biochemistry and Molecular Biology, Jishou University School of Medicine, Jishou, 416000 Hunan China; 3 Department of Pathology and Pathophysiology, Hunan Normal University School of Medicine, Changsha, 410013 Hunan China; 4 School of Information Engineering, Baise University, Baise, 533000 Guangxi China; 5 Department of Epidemiology and Health Statistics, Xiangya Public Health School, Central South University, Changsha, 410078 Hunan China

**Keywords:** Cancer, Computational biology and bioinformatics

## Abstract

In biomedical data mining, the gene dimension is often much larger than the sample size. To solve this problem, we need to use a feature selection algorithm to select feature gene subsets with a strong correlation with phenotype to ensure the accuracy of subsequent analysis. This paper presents a new three-stage hybrid feature gene selection method, that combines a variance filter, extremely randomized tree, and whale optimization algorithm. First, a variance filter is used to reduce the dimension of the feature gene space, and an extremely randomized tree is used to further reduce the feature gene set. Finally, the whale optimization algorithm is used to select the optimal feature gene subset. We evaluate the proposed method with three different classifiers in seven published gene expression profile datasets and compare it with other advanced feature selection algorithms. The results show that the proposed method has significant advantages in a variety of evaluation indicators.

## Introduction

Due to the increase in high-dimensional data and the limited number of samples, the "big P small n" paradigm has become a major challenge in the field of biomedical data mining^1,2^. Especially for microarray profile datasets, the number of genes is much larger than the number of samples, but only a few feature genes are closely related to cancer^3,4^. Feature selection can remove irrelevant and redundant genes, improve the classification and diagnosis rate of cancer, and help to improve the treatment of cancer^5,6^. According to their interaction with classifiers, feature selection methods can be divided into four categories: filter, embedded, wrapper, and hybrid methods^7–10^. The filter method sorts genes according to the correlation of individual genes or the ability to distinguish target categories^11,12^. The embedded method automatically selects the feature gene according to the algorithm^13,14^. It quickly selects the optimal feature gene subset through algorithm training and feature selection at the same time. The wrapper method usually uses the classification model containing a heuristic algorithm and selects the optimal feature subset according to the classification performance^15–17^. Although the wrapper method is lower in computational efficiency than the filter method, its classification performance is usually better than the latter^18^.

The hybrid method is generally a combination of the filter method and wrapper method^19,20^. First, the filter method is used to quickly remove irrelevant features on a large scale and reduce the feature subset. Then, using the wrapper method, the optimal feature gene subset is selected. The hybrid method can combine the computational efficiency of the filter method and the high classification performance of the wrapper method^21^. For example, Su et al. combined the K-S test with CFS and compared it with four advanced methods. The results show that the hybrid method is effective^8^. Elnaz Pashaei^22^, Xiongshi Deng^23^, and Jamshid Pirgazi^24^ also adopted the hybrid method of combining the filter and wrapper method and achieved good results in many public cancer datasets. In recent years, an increasing number of researchers have considered a hybrid method combining filters and wrappers to select features from gene expression data^25^. This paper presents a three-stage hybrid feature selection method: VEW, which combines the filter method and wrapper method. In the first stage, we use a variance filter to filter out genes that do not meet the variance threshold. In the second stage, we use the extremely randomized tree (ERT) algorithm to sort the importance of the gene subsets obtained in the previous stage, and further reduce the subset of feature genes. In the third stage, we input the gene subset obtained in the second stage into the whale optimization algorithm (WOA) to obtain the optimal feature gene subset. Through the analysis and comparison of the experimental results, we verify that the VEW method has obvious advantages in the selection performance of feature genes, the number of selected genes and the calculation time. This paper mainly finds that the three-stage hybrid algorithm combining the filter method and wrapper method has significant performance improvement and is easy to implement.

The rest of this paper is organized as follows: first, we summarize the research work and algorithm principle of the variance filter, ERT, and WOA and introduce the hybrid algorithm VEW in detail. In the results section, based on 7 published cancer gene expression datasets, we compare the VEW method with 11 related feature selection algorithms and other advanced feature selection algorithms. Finally, we summarize the experimental results and future work direction of this paper.

## Methods

### Variance filter

The variance filter is a simple filter method, that can quickly remove low-variance genes with poor classification performance. Michal Marczyk removed redundant feature genes from high-throughput data by an adaptive variance filter, which effectively improved the cancer classification performance^26^. In this paper, we set the variance threshold to 0.05 to quickly screen feature genes in a large range.

### ERT

ERT is similar to the random forest, which is a machine-learning algorithm composed of multiple decision trees. Unlike the random forest, the ERT uses all training samples to obtain each decision tree and forks the decision tree by randomly selecting split nodes. Liang et al.^27^ identified promoters and their strength through feature selection of ERT.

### WOA

**Figure 1:**
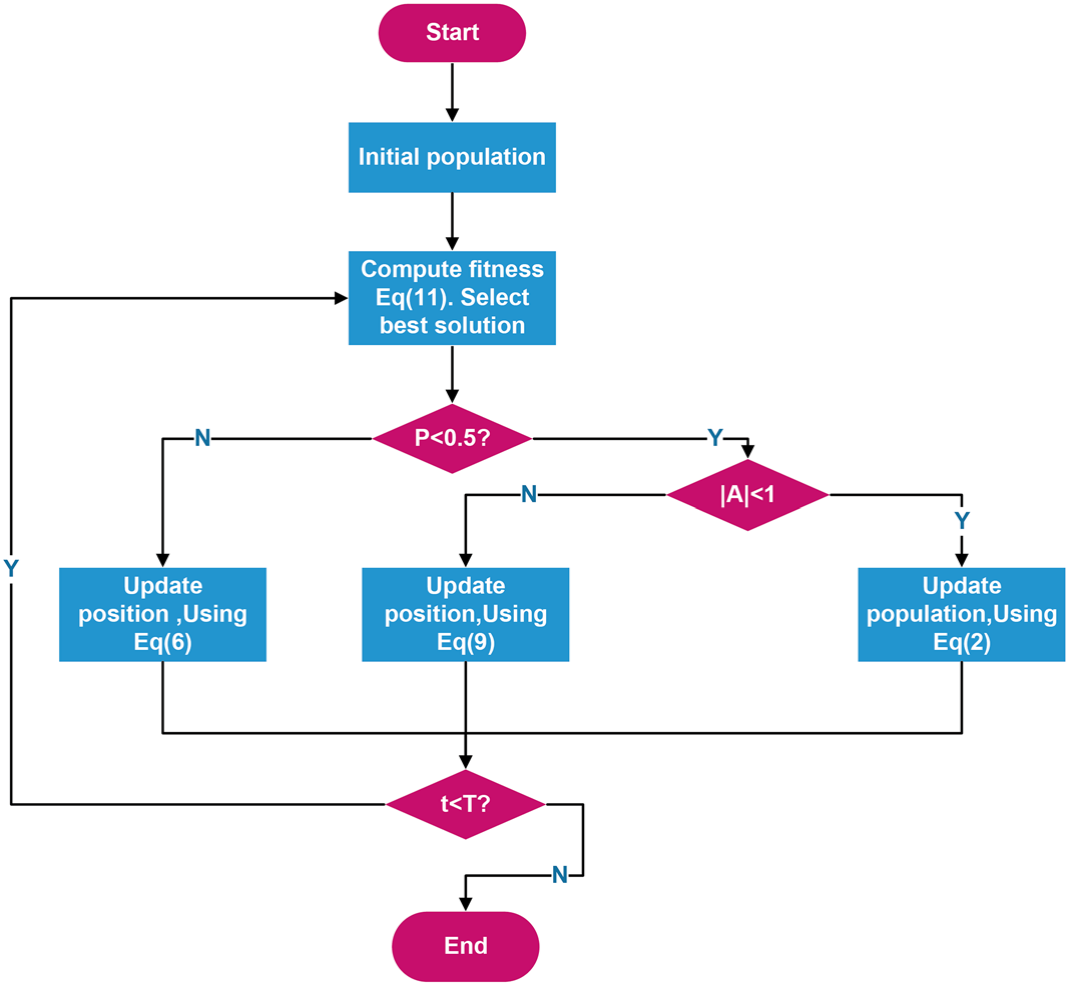
WOA workflow.

Mirjalili (2016) proposed a new swarm intelligence optimization algorithm based on the predatory behaviour of humpback whales: the WOA^28^. The WOA algorithm achieves the goal of optimizing the time by simulating the hunting behaviour of humpback whales in nature, such as whale group search, encirclement, pursuit, and attack of prey. The WOA is divided into the exploration and development stage. In the exploration stage, whales conduct random searches for prey. In the development stage, whales adopt two hunting modes: shrinking enclosure and spiral bubble net. Figure 1 shows the workflow of the WOA. In the development stage, whales hunt in the direction of the current optimal position. In the contraction and encirclement hunting mode, the optimal position in the whale population is set as prey, and other individuals in the population shrink, encircle, and approach the prey. The position update is shown in Formulas (1) and (2):

1 $$D=\left|C{X}_{q,t}-{X}_{i,t}\right|$$

2 $${X}_{i,t+l}={X}_{q,t}-AD$$

where ${X}_{q,t}$ is the current optimal solution, ${X}_{i,t}$ is the current whale individual, and $D$ is the distance between the current whale individual and the current optimal solution.

A is the convergence factor, and C is the disturbance factor. The $A,C$ calculation Formula are shown in (3) and (4):

3 $$A=2a\cdot {rand}_{1}-a$$

4 $$C=2\cdot {rand}_{2}$$

where ${rand}_{1}$ and ${rand}_{2}$ are random numbers between $[\mathrm{0,1}]$. The coefficient $a=2-2t/ T$, and $a$ decreases linearly from 2 to 0. In addition, $t$ is the current iteration number and $T$ is the maximum iteration number.

In the spiral bubble net model, by calculating the distance between whales and prey, whales spit out bubbles in a spiral path to corral prey. The whale spiral position update is shown in (5) and (6):

5 $${D}^{{\prime}}=\left|{X}_{q,t}-{X}_{i,t}\right|$$

6 $${X}_{i,t+l}={X}_{q,t}+{D}^{{\prime}}\cdot {e}^{bl}\mathrm{cos}\left(2\pi l\right)$$

$D^{{\prime}}={|X}_{q,t}-{X}_{i,t}|$ is the distance between an individual whale and prey, and $b$ is the spiral constant, $l\in [-\mathrm{1,1}]$. The whale encircles its prey while spiralling inward. The algorithm selects and distinguishes these two modes through the random variable $p$ and updates the whale position, as shown in Formula (7):

7 $${X}_{i,t+l}\left\{\begin{array}{c}{X}_{q,t}+{D}^{{\prime}}\cdot {e}^{bl}\mathrm{cos}\,\left(2\pi l\right)\, p\ge 0.5\\ {X}_{q,t}-AD\,p<0.5\end{array}\right.$$

In the exploration stage, humpback whales do not know the location of their prey and can only randomly select a whale individual in the population as a target to search for prey. At this time, the random search location update is shown in Formula (8) and (9):

8 $$D=\left|{C\cdot X}_{rand,t}-{X}_{i,t}\right|$$

9 $${X}_{i,t+l}={X}_{rand,t}-AD$$

${X}_{rand,t}$ is the location of randomly selected whales, and $D$ is the distance from humpback whales to randomly selected whales.

### Coding rules

We set the whale individual position as $X=\left\{{x}_{1},\cdots ,{x}_{n}\right\}, { x}_{i}\epsilon [\mathrm{0,1}]$ and convert $X$ to binary position $X^{{\prime}}=\left\{{{x}^{{\prime}}}_{1},\cdots ,{{x}^{{\prime}}}_{n}\right\}, { {x}^{{\prime}}}_{i}\epsilon \{\mathrm{0,1}\}$ with length $n$. Here, ${{x}^{{\prime}}}_{i}=1$ indicates that the feature is selected, and ${{x}^{{\prime}}}_{i}=0$ indicates that the feature is not selected. The WOA algorithm adopts binary encoding as shown in Formula (10):

10 $${x}_{i}^{{\prime}}=\left\{\begin{array}{*{20}l}1 , & \quad if\,rand<{x}_{i}\\ 0 , &\quad otherwise\end{array}\right.$$

where ${x}_{i}$ represents the $i$-dimensional value of an individual whale at position $X$, and $rand$ is a random number between $[\mathrm{0,1}]$.

### Fitness function

The fitness function is used to evaluate the advantages and disadvantages of each feature subset. In this paper, $KNN$ is selected as the fitness function of the classification problem, as shown in Formula (11):

11 $$fitness=\alpha \left(1-{KNN}_{acc}\right)+\left(1-\alpha \right)\cdot \frac{\left|R\right|}{\left|C\right|}$$

where $|R|$ is the length of the selected feature subset and $|C|$ is the total number of features. ${KNN}_{acc}$ is the classification accuracy using the $KNN$ classifier, and $\alpha$ is the weight coefficient. In this paper, we set $\alpha =0.99$.

### VEW

**Figure 2:**
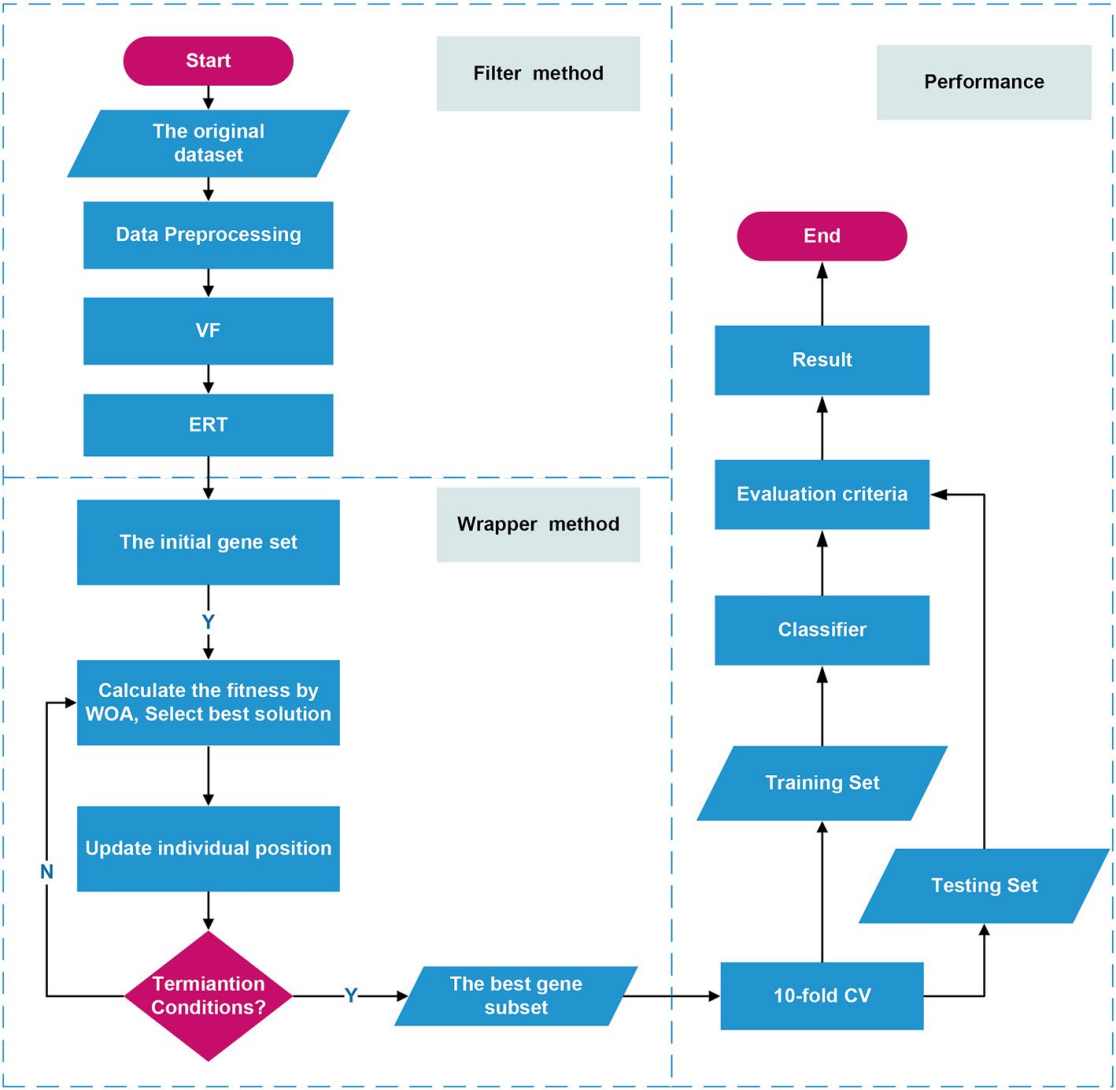
VEW workflow.

In this paper, we propose a three-stage gene selection method: VEW, which combines a variance filter, ERT, and WOA. In the first stage, we use the variance filter method to screen genes and select feature genes that are greater than the variance threshold. In the second stage, we use the ERT to calculate the importance score of each gene, further screen the genes and eliminate the genes with a score of zero. Finally, we use the WOA to obtain the optimal subset of feature genes. The pseudocode code of VEW is shown in Algorithm 1. Figure 2 shows the gene selection process of the VEW algorithm. We also discuss the time complexity of VEW. The time complexity of VEW is mainly composed of two stages. The time complexity of the ERT in the second stage is $O(M\times (mnlogn))$, where $M$ is the number of decision trees, $n$ is the number of genes in the sample and $m$ is the number of feature genes. In the third stage, the time complexity of the WOA is $O(N\times T\times D)$. Here, $N$ is the population size obtained in the second stage, $T$ is the maximum number of iterations and $D$ is the problem size. In the method proposed in this paper, the first two stages involve simple filterings and sorting of gene sets, which are fast and time-consuming, respectively. Because $O(N\times T\times D)\gg O(M\times (mnlogn))$, the time spent by the algorithm is mainly concentrated in the third stage.
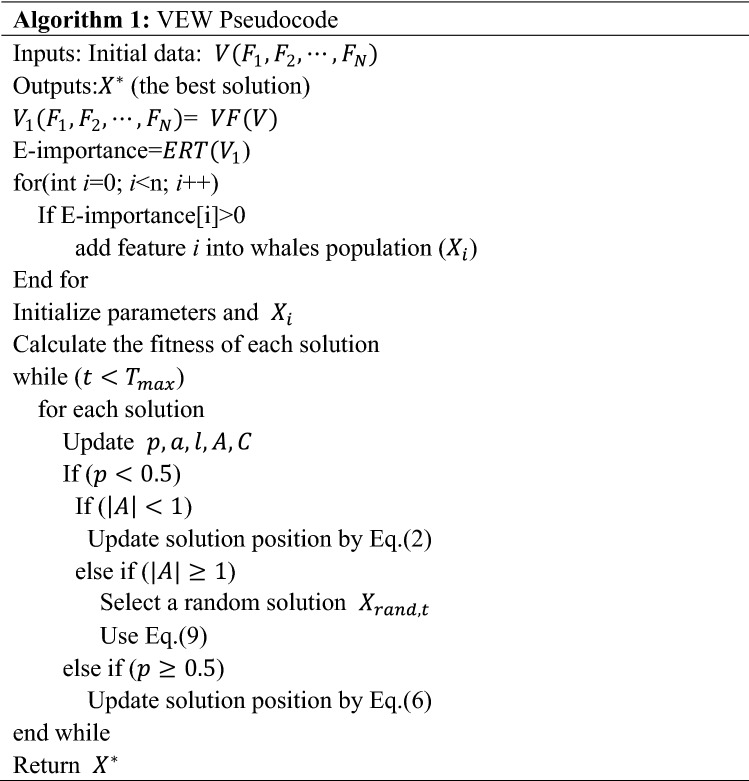

## Results

### Data and parameter setting

To evaluate the performance of each algorithm, seven microarray gene expression profile datasets are used in this paper. All datasets used are from public websites: http://csse.szu.edu.cn/staff/zhuzx/Datasets.Html^29^ and https://github.com/Pengeace/MGRFE-GaRFE^25^. Table S1 in the supplementary material provides a detailed overview of the feature of the seven microarray datasets, including samples, number of genes, and classes. In these datasets, the number of genes ranged from 7129 to 54,675, and the number of samples is less than 300. These datasets include acute lymphoblastic leukemial type L3 (ALL3), gastric 1 (Gas1), type 1 diabetes (T1D), myeloma (Mye), ovarian cancer (Ova), leukemia (Leuk), and mixed-lineage leukemial (MLL). Only the MLL dataset is a three-class dataset, whereas the others are binary. The number of class samples in most datasets is uneven. In data preprocessing, we fill in the missing values with the mean and map the new data values to $[\mathrm{0,1}]$ using the minimum maximum normalization method. All the experimental results in this paper are generated on a PC equipped with a Corei7-8750 CPU, 16 GB of memory, and 2.20 GHz frequency. All algorithms are implemented using Python language and two public package tools; scikit learn and scikit feature. In this paper, we use three different external classifiers to evaluate the performance of each algorithm, namely, the decision tree (DT), support vector machine (SVM), and logistic regression (LR). After tenfold cross-validation of each standard classifier, the classification performance of each algorithm is recorded. The tenfold cross-validation method randomly divides the dataset into 10 parts, nine of which are divided into training sets, and the rest are divided into test sets. We compare the VEW method with 11 different methods and other methods in the literature. The 11 different methods include the T test (T), Wilcoxon test (W), variance filter-univariate feature selection (VU), extremely randomized tree-univariate feature selection (EU), variance filter-extremely randomized tree (VE), variance filter-bat algorithm (VB), extremely randomized tree-bat algorithm (EB), variance filter-firefly algorithm (VF), extremely randomized tree-firefly algorithm (EF), variance filter-whale optimization algorithm (VW) and extremely randomized tree-whale optimization algorithm (EW). Table S2 in the supplementary materials lists the specific parameter values of each algorithm and classifier. All experiments were run independently 10 times, and the average value was taken. Seven evaluation criteria are used to reflect the performance of each algorithm: the number of selected genes, classification accuracy (Acc), precision (Pre), recall rate (Recall), F1-score (F1), standard deviation (SD), and algorithm running time. The calculation formulas for the four important evaluation criteria are as follows:

12 $$Acc=\frac{TN+TP}{P+N}$$

13 $$Precision=\frac{TP}{TP+FP}$$

14 $$Recall=\frac{TP}{TP+FN}$$

15 $$F1=2*\frac{Precision*Recall}{Precision+Recall}$$

The number of positive samples is (P), and the number of negative samples is (N). True positive (TP): the real category of the sample is positive, and the model prediction is also positive. True negative (TN): the real category of the sample is a negative case, and the model prediction is also a negative case. False-positive (FP): the real category of the sample is negative, and the model prediction is positive. False-negative (FN): the real category of the sample is positive and the model prediction is negative. Because the precision, recall, and F1 are for a single class, we assign the same weight to each class and calculate their average values.

### Comparison of performance

We comprehensively compare the VEW method with T, W, VU, EU, VE, VB, EB, VF, EF, VW, and EW. The best performance values in each dataset are highlighted in black bold. Tables 1, 2 and 3 show the performance values of the four evaluation criteria of each algorithm on the three classifiers. It can be seen from Table 1 that on DT, the VEW method has obvious advantages over the other methods. The Acc, Pre, Recall, and F1 winning times are 7, 6, 7, and 6 times, respectively. The average Acc on seven datasets reaches 86.47%, which is significantly better than the other nine methods, with 100% Acc achieved on the ovarian dataset. As shown in Table 2, on the SVM, the winning times of the VEW method on the four evaluation criteria is 6, and the Acc is 100% on the ovarian and leukemia datasets. Moreover, the average Acc reaches 89.00%. As shown in Table 3, on LR, the number of winning times of the VEW on the four evaluation criteria is 6 and reaches 100% on both the leukemia and ovarian datasets. The average Acc of VEW was significantly higher than that of the other nine methods and reaches a maximum of 89.58%. In summary, compared with other methods, VEW has obvious advantages in Acc, pre, recall, and F1, especially in DT, and achieves the highest average Acc in LR. This also proves that the hybrid method proposed in this paper can effectively improve the performance of each index. Table S3 in the supplementary materials lists the number of genes selected by each algorithm in the seven datasets. From the results, the average number of genes selected by the VEW method is the lowest. VW and EW selects fewer genes than VEW in three of the datasets, but combined with other indicators, we find that this advantage comes at the expense of other performances. In addition, in most datasets, the number of genes selected by the VEW method is only 1/4 to 1/250 of that of other comparative methods. The above experiments prove that VEW can better combine the advantages of various methods and select fewer feature genes without sacrificing performance. To further verify the performance advantages of the VEW method, we compare it with other advanced algorithms in the literature. Table 4 lists the comparison results between the proposed algorithm and other literature methods, where "\" indicates the lack of experimental data. It can be seen from the results in Table 4 that compared with other advanced algorithms, the VEW method also has certain competitiveness in Acc.

**Table 1:** Performance comparison of algorithms on classifier DT.

Data	Measure	T	W	VU	EU	VE	VB	EB	VF	EF	VW	EW	VEW
ALL3	Acc	76	76	64	69	65.6	64	71.20	67	61	68	65.6	**78.67**
	SD	0	0	0	5.03	3.58	3.27	8.20	10	5.03	8.00	6.07	2.31
	Pre	78.18	71.48	55.27	64.15	57.98	58.82	68.83	67.76	59.13	64.21	64.02	**78.90**
	Recall	76	76	64	69	65.6	64	71.20	67	61	68	65.06	**78.67**
	F1	75.40	71.14	59.32	66.18	61.47	61.19	68.22	65.06	59.93	65.77	64.72	**75.86**
Gas1	Acc	86.21	79.31	86.21	89.66	88.80	90.81	83.91	85.06	87.36	78.45	81.04	**91.38**
	SD	0	0	0	3.45	3.30	1.99	5.27	1.99	2.00	7.65	8.21	1.88
	Pre	86.97	79.97	86.21	90.02	89.04	90.95	84.27	85.35	87.64	80.12	81.88	**91.47**
	Recall	86.21	79.31	86.21	89.66	88.80	90.81	83.91	85.06	87.36	78.45	81.04	**91.38**
	F1	86.17	79.26	86.21	89.65	88.79	90.81	83.89	85.03	87.33	78.19	80.94	**91.37**
T1D	Acc	52.38	66.67	61.9	57.14	60.71	57.14	47.62	55.95	54.76	57.14	52.38	**70.24**
	SD	0	0	0	8.69	8.13	3.89	8.69	10.56	9.12	11.00	11.17	8.13
	Pre	52.38	66.67	63.17	57.49	60.92	57.30	47.44	56.61	55.21	57.57	52.81	**70.34**
	Recall	52.38	66.67	61.90	57.14	60.71	57.14	47.62	55.95	54.76	52.38	52.38	**70.24**
	F1	52.38	66.51	60.07	56.66	60.61	55.51	47.21	55.28	54.05	56.91	51.66	**70.11**
Mye	Acc	74.29	71.43	62.86	66.47	71.43	70	69.29	74.29	65.72	67.86	70.29	**75**
	SD	0	0	0	2.78	6.17	5.95	6.34	6.17	4.04	8.84	7.17	4.28
	Pre	76.27	75.51	73.43	71.50	74.69	75.90	**77.62**	79.95	73.20	75.42	76.73	77.44
	Recall	74.29	71.43	62.86	66.43	71.43	70.00	69.29	74.29	65.72	67.86	70.29	**75**
	F1	75.24	73.32	67.36	68.81	72.97	72.05	72.60	**76.49**	68.99	70.75	73.04	76.13
Ovarian	Acc	86.27	90.20	100	90.20	100	98.04	93.14	96.57	93.14	96.57	93.63	**100**
	SD	0	0	0	0	0	0	1.13	1.88	2.53	2.94	4.04	0
	Pre	86.23	90.41	100	90.34	100	98.10	93.29	96.68	93.64	97.04	93.88	**100**
	Recall	86.27	90.20	100	90.20	100	98.04	93.14	96.57	93.14	96.57	93.63	**100**
	F1	85.90	89.93	100	89.98	100	98.02	93.02	96.55	92.92	96.62	93.47	**100**
Leuk	Acc	93.33	86.67	93.33	93.33	93.33	91.66	85.00	95	90.88	81.67	83.34	**96.66**
	SD	0	0	0	0	0	3.33	3.34	6.38	3.17	3.34	3.85	3.85
	Pre	93.94	88.89	93.94	93.94	93.94	93.08	86.00	95.71	92.18	82.60	84.63	**96.97**
	Recall	93.33	86.67	93.33	93.33	93.33	91.66	85.00	95	90.88	81.67	83.34	**96.66**
	F1	93.12	85.61	93.12	93.12	93.12	91.60	84.32	94.68	90.47	80.61	83.26	**96.56**
MLL	Acc	73.33	66.67	93.33	86.66	93.33	89.99	90	88.33	76.67	78.34	66.67	**93.33**
	SD	0	0	0	12.17	0	11.55	8.61	6.38	8.61	15.75	9.43	0
	Pre	73.89	66.67	94.44	90.46	94.44	91.32	91.79	89.84	79.74	80.95	66.99	**94.44**
	Recall	73.33	66.67	93.33	86.66	93.33	89.99	90.00	88.33	76.67	78.34	66.67	**93.33**
	F1	73.13	65.15	93.27	86.91	93.27	89.62	89.25	88.16	76.38	78.77	65.97	**93.27**
Winner	Acc	0	0	0	0	0	0	0	0	0	0	0	**7**
	Pre	0	0	0	0	0	0	1	0	0	0	0	**6**
	Recall	0	0	0	0	0	0	0	0	0	0	0	**7**
	F1	0	0	0	0	0	0	0	1	0	0	0	**6**
Mean	Acc	77.40	76.71	80.23	78.92	81.89	80.23	77.17	80.31	75.65	75.43	73.28	**86.47**

**Table 2:** Performance comparison of algorithms on classifier SVM.

Data	Measure	T	W	VE	EU	VE	VB	EB	VF	EF	VW	EW	VEW
ALL3	Acc	76	72	76	76	76	76	76	76	76	71.2	69.6	**81.33**
	SD	0	0	0	0	0	0	0	0	0	9.12	8.76	6.11
	Pre	57.76	66.73	57.76	57.76	57.76	57.76	57.76	57.76	57.76	62.45	63.83	**81.76**
	Recall	76	72	76	76	76	76	76	76	76	71.2	69.60	**81.33**
	F1	65.64	68.36	65.64	65.64	65.64	65.64	65.64	65.64	65.64	65.76	65.33	**77.20**
Gas1	Acc	93.10	93.10	93.10	93.10	93.10	93.10	93.10	93.10	93.10	87.07	90.52	**93.96**
	SD	0	0	0	0	0	0	0	0	0	7.65	5.17	1.73
	Pre	93.10	93.10	93.10	93.10	93.10	93.10	93.10	93.10	93.10	87.72	90.57	**94.22**
	Recall	93.10	93.10	93.10	93.10	93.10	93.10	93.10	93.10	93.10	87.07	90.52	**93.96**
	F1	93.10	93.10	93.10	93.10	93.10	93.10	93.10	93.10	93.10	86.99	90.52	**93.95**
T1D	Acc	61.90	57.14	57.14	57.14	57.14	52.38	59.52	61.9	60.71	53.57	57.14	**67.86**
	SD	0	0	0	0	3.89	0	6.15	3.89	4.56	7.14	3.37	7.15
	Pre	62.70	58.38	58.38	56.66	57.85	52.91	59.79	62.17	61.12	53.87	54.62	**68.50**
	Recall	61.90	57.14	57.14	57.14	57.14	52.38	59.52	61.90	60.71	53.57	54.28	**67.86**
	F1	61.73	56.55	56.55	56.99	56.89	52.16	59.47	61.82	60.67	53.36	53.52	**67.59**
Mye	Acc	77.14	80	71.43	82.86	76.43	77.14	77.86	78.57	80	80.72	74.86	**84.29**
	SD	0	0	0	0	2.73	2.33	4.88	4.95	0	6.33	10.18	3.69
	Pre	72.32	78.23	71.43	79.76	73.58	74.90	74.79	74.90	75.48	77.71	77.23	**85.71**
	Recall	77.14	80	71.43	82.86	76.43	77.14	77.86	78.57	80	80.72	74.86	**84.29**
	F1	74.65	79.05	71.43	80.99	74.96	75.98	76.18	76.56	77.62	78.75	75.00	**84.88**
Ovarian	Acc	**100**	**100**	**100**	**100**	**100**	**100**	**100**	**100**	**100**	97.55	**100**	**100**
	SD	0	0	0	0	0	0	0	0	0	4.90	0	0
	Pre	**100**	**100**	**100**	**100**	**100**	**100**	**100**	**100**	**100**	97.53	**100**	**100**
	Recall	**100**	**100**	**100**	**100**	**100**	**100**	**100**	**100**	**100**	96.77	**100**	**100**
	F1	**100**	**100**	**100**	**100**	**100**	**100**	**100**	**100**	**100**	97.53	**100**	**100**
Leuk	Acc	93.33	86.67	**100**	**100**	**100**	**100**	**100**	**100**	**100**	80	81.67	**100**
	SD	0	0	0	0	0	0	0	0	0	9.43	14.78	0
	Pre	94.13	90.48	**100**	**100**	**100**	**100**	**100**	**100**	**100**	84.06	83.14	**100**
	Recall	93.33	86.67	**100**	**100**	**100**	**100**	**100**	**100**	**100**	80	82.67	**100**
	F1	93.24	87.04	**100**	**100**	**100**	**100**	**100**	**100**	**100**	80.51	82.01	**100**
MLL	Acc	**100**	**100**	93.33	96.66	93.33	88.33	**100**	93.33	91.66	65	68.33	95.55
	SD	0	0	0	3.85	0	6.38	0	0	3.33	6.38	11.38	3.85
	Pre	**100**	**100**	94.44	97.22	94.44	89.03	**100**	94.44	94.50	68.01	70.68	96.29
	Recall	**100**	**100**	93.33	96.66	93.33	88.33	**100**	93.33	91.66	65	68.33	95.55
	F1	**100**	**100**	93.27	96.63	93.27	88.25	**100**	93.27	91.62	65.47	68.40	95.51
Winners	Acc	2	2	2	2	2	2	3	2	2	0	1	**6**
	Pre	2	2	2	2	2	2	3	2	2	0	1	**6**
	Recall	2	2	2	2	2	2	3	2	2	0	1	**6**
	F1	2	2	2	2	2	2	3	2	2	0	1	**6**
Mean	Acc	85.92	84.13	84.43	86.54	85.14	83.85	86.64	86.13	85.92	76.44	77.45	**89.00**

**Table 3:** Performance comparison of algorithms on classifier LR.

Data	Measure	T	W	VE	EU	VE	VB	EB	VF	EF	VW	EW	VEW
ALL3	Acc	56	60	76	74	72	63	72.8	74	75	58.40	66.40	**81.33**
	SD	0	0	0	2.31	4	3.83	5.22	2.31	3.83	10.81	4.56	6.11
	Pre	58.32	60	71.48	60.81	67.91	58.57	66.54	66.67	72.28	63.83	63.31	**79.09**
	Recall	56	60	76	74	72	63	72.8	74	75	58.00	66.40	**81.33**
	F1	57.10	60	71.14	66.01	68.98	60.61	67.18	68.57	70.47	60.23	64.31	**78.72**
Gas1	Acc	86.21	79.31	93.10	93.10	93.10	93.10	93.10	93.10	93.10	83.62	80.17	**93.10**
	SD	0	0	0	0	0	0	0	0	0	7.65	10.29	0
	Pre	86.97	79.97	93.10	93.10	93.10	93.10	93.10	93.10	93.10	83.86	80.80	**93.30**
	Recall	86.21	79.31	93.10	93.10	93.10	93.10	93.10	93.10	93.10	83.62	80.17	**93.10**
	F1	86.17	79.26	93.10	93.10	93.10	93.10	93.10	93.10	93.10	83.61	79.87	**93.10**
T1D	Acc	57.14	57.14	71.43	60.71	63.10	65.48	60.71	60.71	60.71	55.95	54.28	**78.57**
	SD	0	0	0	4.56	4.56	2.39	4.56	5.99	4.56	10.56	5.43	2.75
	Pre	57.40	57.40	71.43	60.92	63.14	65.70	61.16	61.07	61.12	57.28	54.62	**80.10**
	Recall	57.14	57.14	71.43	60.71	63.10	65.48	60.71	60.71	60.71	55.95	54.28	**78.57**
	F1	57.14	57.14	71.43	60.71	63.01	65.48	60.57	60.44	60.67	54.49	53.52	**78.10**
Mye	Acc	74.29	74.29	68.57	72.15	65.72	62.86	75	75	69.99	60.71	72	**80.72**
	SD	0	0	0	2.74	3.30	7.38	4.28	7.51	5.47	7.87	3.13	4.28
	Pre	79.76	79.76	70.94	75.71	74.12	73.43	76.60	78.97	76.09	78.19	77.09	**85.83**
	Recall	74.29	74.29	68.57	72.15	65.72	62.85	75	75	69.99	60.71	72	**80.72**
	F1	76.58	76.58	69.73	73.82	69.37	67.37	75.71	76.61	72.69	66.37	74.13	**82.52**
Ovarian	Acc	**100**	**100**	**100**	**100**	**100**	**100**	**100**	**100**	**100**	96.57	99.02	**100**
	SD	0	0	0	0	0	0	0	0	0	2.94	1.96	0
	Pre	**100**	**100**	**100**	**100**	**100**	**100**	**100**	**100**	**100**	96.65	99.12	**100**
	Recall	**100**	**100**	**100**	**100**	**100**	**100**	**100**	**100**	**100**	96.57	99.02	**100**
	F1	**100**	**100**	**100**	**100**	**100**	**100**	**100**	**100**	**100**	96.58	99.03	**100**
Leuk	Acc	86.67	94.29	**100**	**100**	**100**	**100**	**100**	**100**	**100**	73.33	86.66	**100**
	SD	0	0	0	0	0	0	0	0	0	18.86	15.40	0
	Pre	90.48	94.60	**100**	**100**	**100**	**100**	**100**	**100**	**100**	75.42	90.64	**100**
	Recall	86.67	94.29	**100**	**100**	**100**	**100**	**100**	**100**	**100**	74.33	86.66	**100**
	F1	87.04	94.32	**100**	**100**	**100**	**100**	**100**	**100**	**100**	73.80	86.97	**100**
MLL	Acc	**100**	**100**	**100**	98.33	**100**	**100**	96.66	96.66	98.33	76.67	78.33	93.33
	SD	0	0	0	3.34	0	0	3.85	3.85	3.34	11.55	6.39	0
	Pre	**100**	**100**	**100**	98.61	**100**	**100**	97.22	97.22	98.61	79.62	82.25	94.44
	Recall	**100**	**100**	**100**	98.33	**100**	**100**	96.66	96.66	98.33	76.67	78.33	93.33
	F1	**100**	**100**	**100**	98.32	**100**	**100**	96.63	96.63	98.32	76.47	77.96	93.27
Winners	Acc	2	2	3	2	3	3	2	2	2	0	0	**6**
	Pre	2	2	3	2	3	3	2	2	2	0	0	**6**
	Recall	2	2	3	2	3	3	2	2	2	0	0	**6**
	F1	2	2	3	2	3	3	2	2	2	0	0	**6**
Mean	Acc	80.04	80.72	87.01	85.47	84.85	83.49	85.47	85.64	85.30	72.18	76.69	**89.58**

**Table 4:** Comparison between the VEW and other advance methods in Acc.

Methods	ALL3	Gas1	T1D	Mye	Ovarian	Leuk	MLL
McOne^29^	80	91	70	83	\	98	\
RRF^29^	79	91	72	80	\	92	\
CFS^23^	\	\	\	70.52	80.63	75.89	\
ISFLA^37^	\	\	\	\	\	95.84	92.62
WOASAT^37^	\	\	\	\	\	92.50	92.62
MPSO^37^	\	\	\	\	\	91.71	90.64
FCSVM-RFE^23^	\	\	\	64.31	86.55	93.21	\
Xgboost-MOGA^23^	\	\	\	81.54	99.22	98.57	\
VEW	81.33	93.96	78.57	84.29	100	100	95.55

### Comparison of running time

We analyse the running time of all algorithms, and Table 5 lists the average running time of each algorithm on each dataset. It can be seen from the results that the EF method has the longest running time and the VE method has the shortest average running time. The running time of VEW is less than that of T, W, VB, EB, VF, EF, VW, and EW and more than that of VU, EU, and VE. According to the previous analysis results, other comparison methods are significantly lower than VEW in terms of Acc, the number of selected genes, etc. This shows that VEW can improve other performances and shorten the overall running time through the hybrid method.

**Table 5:** Comparison of the running time $(10s)$ between the VEW and other methods.

Data	T	W	VU	EU	VE	VB	EB	VF	EF	VW	EW	VEW
ALL3	31.70	28.18	7.96	7.51	**7.11**	77.31	65.95	672.18	688.28	38.73	39.75	29.07
Gas1	195.42	97.86	7.27	5.90	**4.83**	106.37	58.54	504.71	521.85	48.24	32.41	19.57
T1D	144.42	134.70	19.29	**9.47**	11.46	346.48	78.48	575.54	588.75	142.19	35.31	36.44
Mye	5.39	3.89	2.47	4.25	**2.59**	29.86	46.12	556.73	511.36	24.93	32.58	20.34
Ova	90.82	84.44	6.08	7.15	**4.50**	72.06	84.90	857.12	888.35	42.34	49.09	23.74
Leuk	18.72	17.71	7.25	**3.59**	4.06	187.53	39.12	261.30	278.09	91.70	20.45	20.66
MLL	34.97	32.13	7.90	**4.35**	5.16	148.20	45.28	335.22	338.23	62.35	23.99	23.36
Mean	74.49	56.99	8.32	6.03	**5.67**	138.26	59.77	537.54	544.99	64.35	33.37	24.74

### Biological inferences

Due to the randomness of the VEW method, multiple results with the same performance but different feature genes may be obtained in multiple experiments. We adopt the following principles to solve this problem: (1). The results with high Acc in multiple classifiers are comprehensively selected. (2). When Acc is the same, a subset of feature genes with a small number is preferentially selected. (3). When the numbers of Acc and feature genes are the same, the subset of genes with the highest frequency is selected. Table 6 lists the number of optimal gene subsets, probe/UniProt ID and average Acc on different classifiers selected by VEW in each dataset after 10 independent runs. To test the effectiveness of VEW in the selection of cancer-related biomarkers, we perform biological inference on the selected best subset of genes (partial genes) in three of the datasets. Tables S4–S6 in the supplementary materials list the probe/UniProt ID, gene name and gene function description corresponding to the best gene subset selected by VEW on the three datasets.

**Table 6:** Optimal subset of genes selected by the VEW.

Data	Number	Probe/uniprot ID	DT	SVM	LR
ALL3	9	1011_s_at,1077_at,34329_at,34582_at 35530_f_at, 37901_at, 38433_at 38525_at, 41801_at	80	88	88
Gas1	5	56256_at,202954_at,210066_s_at 213905_x_at,215901_at	96.55	96.55	96.55
T1D	7	210649_s_at,215037_s_at,215728_s_at 219010_at,240824_at,1554899_s_at 1570229_at	66.67	76.19	80.95
Mye	10	1037_at,1076_at,1103_at,1184_at 1190_at,120_at,1441_s_at,1461_at 1488_at,1518_at	80	80	82.86
Ova	4	MZ2.7921478,MZ2.8548732, MZ224.37109, MZ555.74254	100	100	100
Leuk	7	M27891_at,M63138_at,M84526_at,S70609_at,D28235_s_at,U75276_s_at U47686_s_at	100	100	100
MLL	10	X31637_s_at,X35484_at,X40300_g_at X33423_g_at,X33852_at,X34833_at X1389_at,X1395_at,X963_at,X755_at	93.33	100	93.33

Forgione et al. found that KMT2A is associated with ALL and that KMT2A rearrangement is a driver of highly pathogenic leukemia^30^. FASN is the only human lipogenic enzyme that can be used for de novo fatty acid synthesis and is highly expressed in cancer cells. Reducing FASN expression can make ALL cells sensitive to differentiation therapy^31^. Vojta et al. determined MGAT5B is widely associated with a variety of cancer types, including gastric cancer, and may have potential value for disease prognosis^32^. Rosenblum et al. found that DPP7 plays an important role in regulating peptide hormone signalling and can serve as an emerging target for a variety of cancers including myeloma^33^. ITGAX is closely related to the treatment of multiple cancers, but its correlation with myeloma needs further study^34^. Gao et al. found that MUC1 is a potential target for developing drugs for myeloma patients, and MUC1 based cancer vaccines can effectively prevent cancer progression and metastasis^35^. Similarly, PA2G4 plays an important role in the progression and spread of myeloma and can serve as a potential new therapeutic target for myeloma^36^. The above results show the validity of VEW in biological inference and the practicability of the method proposed in this paper. Of all the evaluation criteria, Acc was the most important, so we tested the performance of the VEW method in the dataset when $\alpha$ took different values. As shown in Table S7, when $\alpha =0.99$, the algorithm performance was the best. Therefore, we set $\alpha =0.99$.

## Conclusion

The purpose of VEW is to select effective feature genes from high-dimensional gene expression data. Unlike other similar methods, VEW is a three-stage hybrid method that combines the three constitutive methods well. We quickly screen feature genes in a large range through a variance filter and ERT and then accurately screen them in a small range through a WOA. This improves performance and reduces time consumption. The results in Tables S4–S6 show that our method can select important genes related to a tumor in multiple datasets, and the results of other researchers also verify the effectiveness and practicability of genes selected by the VEW method from a medical perspective. The results in Tables 1, 2, 3, 4, 5 show that VEW significantly improves performance while reducing run time. The number of genes selected by VEW on all datasets is no more than 10, and the Acc reaches 100% on the ovarian and leukemia datasets; the average Acc on multiple datasets also reaches 89.58%. Compared with other advanced algorithms, VEW has obvious advantages in the number of gene selections, Acc, Precision, Recall, F1, and running time.

As shown in Table S7, we also test the performance value of the VEW method on different datasets when $\alpha$ takes different values, which proves the rationality of our $\alpha$ value. Because the variance filter is simple and efficient, we first use it to filter out redundant genes and use the ERT in the second phase of VEW, which can further narrow the scope of gene screening, increase the randomness of the screening process, and avoid falling into local optimization. The results of the basic WOA in the third stage also show that our idea can significantly improve the overall algorithm performance. We believe that the addition of the three-stage hybrid algorithm of the ERT is the key reason for the performance improvement. The ERT increases the randomness of the overall algorithm and further sorts and filters the gene subset, which also increases the screening accuracy of the whale optimization algorithm. However, the methods proposed in this paper also have many limitations. For example, the basic WOA has the disadvantages of low accuracy, slow convergence speed, and easy trapping in local optima. In addition, the filtering method in the first stage needs to select the better one to improve the performance of the overall algorithm. In future research, we can further select better filter and wrapper methods and combine them with ERT to form a new three-stage hybrid algorithm to improve the performance of the overall algorithm.

## Supplementary Information

Supplementary Information.

## Data Availability

All datasets used are from public websites: http://csse.szu.edu.cn/staff/zhuzx/Datasets.Html and https://github.com/Pengeace/MGRFE-GaRFE.

## Abbreviations

ERT: Extremely randomized tree
WOA: Whale optimization algorithm
VEW: Variance filter-Extremely randomized tree-Whale optimization algorithm
ALL3: Acute lymphoblastic leukemia type L3
Gas1: Gastric1
T1D: Type1 diabetes
Mye: Myeloma
Ova: Ovarian cancer
Leuk: Leukemia
MLL: Mixed-lineage leukemia
DT: Decision Tree
SVM: Support Vector Machine
LR: Logistic Regression
T: T-test
W: Wilcoxon-test
VU: Variance filter-univariate feature selection
EU: Extremely randomized tree-univariate feature selection
VE: Variance filter-extremely randomized tree
VB: Variance filter-bat algorithm
EB: Extremely randomized tree-bat algorithm
VF: Variance filter-firefly algorithm
EF: Extremely randomized tree-firefly algorithm
VW: Variance filter-whale optimization algorithm
EW: Extremely randomized tree-whale optimization algorithm
Acc: Classification accuracy
Pre: Precision
Recall: Recall rate
F1: F1-Score
SD: Standard deviation
TP: True positive
TN: True negative
FP: False positive
FN: False negative
