# Supplementary material for: A new hybrid algorithm for three-stage gene selection based on whale optimization
Source: Sci Rep. 2023 Mar 7;13:3783. doi: 10.1038/s41598-023-30862-y (PMC9992521; doi:10.1038/s41598-023-30862-y)
Supplement: Supplementary file 1 — Supplementary Information. [file 41598_2023_30862_MOESM1_ESM.docx]

**Supplementary information**

**Table S1** Microarray dataset

| No | Dataset | Samples | Genes | Class |
| --- | --- | --- | --- | --- |
| 1 | ALL3 | 125(1:24,2:101) | 12625 | 2 |
| 2 | Gastric1 | 144(1:72,2:72) | 22283 | 2 |
| 3 | T1D | 101(1:57,2:44) | 54675 | 2 |
| 4 | Myeloma | 173(1:137,2:36) | 12625 | 2 |
| 5 | Ovarian | 253(Normal:91,Cancer:162) | 15154 | 2 |
| 6 | Leukemia | 72(ALL:47,AML:25) | 7129 | 2 |
| 7 | MLL | 72(ALL:24,MLL:20,AML:28) | 12582 | 3 |

**Table S2** Parameters of each algorithm and classifier

| Algorithm | Parameter |
| --- | --- |
| T | $\alpha=0.0001$ |
| W | $\alpha=0.0001$ |
| Variance Filter | Variance Threshold (VT):0.05 |
| ERT | nEstimators:100, minSamplesLeaf:20, maxLeafNodes:10 |
| UFS | SelectPercentile:50% |
| WOA | Population size n:30, number of generations T:100, b=1,$\alpha=0.99$ |
| BA | Population size n:30, number of generations T :100, maximum loudness A:2, maximum pulse rate r:1 |
| FA | Population size n:30, number of generations T :100, light amplitude L:1, absorbtion coefficient C:1 |
| DT | Random state:0, max depth:8 |
| SVM | regularization parameter C:1.0, Radial Basis Function |
| LR | regularization parameter C:1.0, Regularization selection parameters: l2 |

**Table S3** Comparison between the VEW based on the number of selected genes and other methods

| Data | Measure | T | W | VU | EU | VE | VB | | EB | VF | EF | VW | EW | VEW |
| --- | --- | --- | --- | --- | --- | --- | --- | --- | --- | --- | --- | --- | --- | --- |
| ALL3 | Avg | 267 | 120 | 340 | 371.17 | 265.86 | | 312.43 | 351.17 | 356.5 | 364.33 | 31.67 | 95.29 | **8.88** |
|  | SD | 0 | 0 | 0 | 8.66 | 12.86 | | 14.25 | 9.56 | 10.03 | 10.48 | 12.86 | 41.78 | 6.90 |
| Gas1 | Avg | 11118 | 9979 | 598 | 259.29 | 165.67 | | 516.5 | 201.17 | 213 | 209.67 | **2** | 2 | 5.17 |
|  | SD | 0 | 0 | 0 | 5.41 | 7.92 | | 8.87 | 9.87 | 10.49 | 11.38 | 0.89 | 1.55 | 0.75 |
| T1D | Avg | 1324 | 456 | 3344 | 350.86 | 765.29 | | 3242.4 | 324.83 | 322 | 330.57 | 30.83 | 55.33 | **8.50** |
|  | SD | 0 | 0 | 0 | 2.91 | 10.29 | | 19.4 | 14.61 | 4.38 | 19.79 | 12.09 | 41.28 | 3.00 |
| Mye | Avg | 49 | 32 | 40 | 122.17 | 32.29 | | 38.29 | 110.33 | 116.83 | 116.5 | 32 | 125.2 | **8.67** |
|  | SD | 0 | 0 | 0 | 2.48 | 2.29 | | 5.99 | 10.93 | 7.78 | 11.64 | 18.84 | 30.04 | 1.75 |
| Ova | Avg | 5235 | 5169 | 276 | 438.33 | 103.17 | | 246.17 | 377.33 | 380.5 | 371 | 6.67 | 7.33 | **5.50** |
|  | SD | 0 | 0 | 0 | 13.85 | 3.43 | | 9.54 | 12.32 | 15.91 | 27.51 | 8.16 | 4.50 | 1.00 |
| Leuk | Avg | 511 | 402 | 1507 | 182.67 | 327.83 | | 1420.83 | 140 | 148.83 | 148.5 | **3** | 4.33 | 6.75 |
|  | SD | 0 | 0 | 0 | 5.39 | 19.47 | | 8.28 | 5.37 | 9.68 | 9.52 | 1.55 | 2.73 | 0.96 |
| MLL | Avg | 563 | 321 | 1080 | 247 | 434 | | 1014.67 | 211.83 | 210.5 | 206.5 | 13.5 | **5.83** | 7.40 |
|  | SD | 0 | 0 | 0 | 3.22 | 10.33 | | 14.09 | 4.07 | 13.07 | 8.31 | 7.99 | 2.71 | 2.97 |
| Ave | | 2723.8 | 2354.2 | 1026.4 | 281.64 | 299.16 | | 970.18 | 245.24 | 249.74 | 249.58 | 17.10 | 42.19 | **7.27** |

**Table S4** Description of ALL3 genes selected by the VEW

| Probe/uniprot ID | Gene | Description |
| --- | --- | --- |
| 1011_s_at | KMT2A | lysine methyltransferase 2A |
| 1077_at | TTC17 | tetratricopeptide repeat domain 17 |
| 34329_at | rbcL | rbcL protein |
| 34582_at | ASPWEDRAFT_34582 | uncharacterized protein |
| 38525_at | L | polymerase protein |
| 41801_at | FASN | fatty acid synthase |

**Table S5** Description of Gas1 genes selected by the VEW

| Probe/uniprot ID | Gene | Description |
| --- | --- | --- |
| 56256_at | MGAT5B | alpha-1,6-mannosylglycoprotein 6-beta-N-acetylglucosaminyltransferase B |
| 202954_at | purL | phosphoribosylformylglycinamide synthetase |
| 210066_s_at | trmB | tRNA m(7)G46 methyltransferase |
| 215901_at | NS | non-structural protein NS1 |

**Table S6** Description of Myeloma genes selected by the VEW

| Probe/uniprot ID | Gene | Description |
| --- | --- | --- |
| 1037_at | CEMP1 | cementum protein 1 |
| 1076_at | DPP7 | dipeptidyl peptidase 7 |
| 1103_at | ITGAX | integrin subunit alpha X |
| 1184_at | MUC1 | mucin 1, cell surface associated |
| 1190_at | PA2G4 | proliferation-associated 2G4 |
| 120_at | PCMT1 | protein-L-isoaspartate O-methyltransferase |
| 1441_s_at | COL4A1 | collagen type IV alpha 1 chain |
| 1461_at | FOXA1 | forkhead box A1 |
| 1488_at | SYNJ1 | synaptojanin 1 |
| 1518_at | ITGB4 | integrin subunit beta 4 |

**Table S7** Comparison of Acc between different α values in VEW

| Dataset | Measure | 0.1 | 0.2 | 0.3 | 0.4 | 0.5 | 0.6 | 0.7 | 0.8 | 0.9 | 0.99 |
| --- | --- | --- | --- | --- | --- | --- | --- | --- | --- | --- | --- |
| ALL3 | DT | 76 | 76 | 52 | 72 | 64 | 74 | 60 | 72 | 64 | **78.67** |
|  | SVM | 72 | 72 | 68 | 72 | 60 | 76 | 76 | 64 | 76 | **81.33** |
|  | LR | 56 | 56 | 64 | 72 | 56 | 60 | 44 | 60 | 72 | **81.33** |
| Gas1 | DT | 75.86 | 89.66 | 75.86 | 75.86 | 79.31 | 68.97 | 79.31 | 72.41 | 82.76 | **91.38** |
|  | SVM | 93.10 | 93.55 | 82.76 | 82.76 | 93.10 | 86.21 | 89.66 | 82.76 | 93.10 | **93.96** |
|  | LR | 86.21 | 82.76 | 89.66 | 89.66 | 82.76 | 86.21 | 89.66 | 89.66 | 86.21 | **93.10** |
| T1D | DT | 42.86 | 57.14 | 47.62 | 47.62 | 61.90 | 66.67 | 52.38 | 52.38 | 57.14 | **70.24** |
|  | SVM | 47.62 | 52.38 | 61.90 | 57.14 | 42.86 | 47.62 | 47.62 | 47.62 | 57.14 | **67.86** |
|  | LR | 47.62 | 61.90 | 47.62 | 57.14 | 47.62 | 61.90 | 57.14 | 57.14 | 61.90 | **78.57** |
| Myeloma | DT | 65.71 | 74.29 | 71.43 | 68.57 | 65.71 | 80 | 68.57 | 68.57 | 72.86 | **75** |
|  | SVM | 71.43 | 74.29 | 82.71 | 77.14 | 71.43 | 80 | 71.43 | 74.29 | 78.57 | **84.29** |
|  | LR | 68.57 | 65.71 | 68.57 | 60 | 62.86 | 71.43 | 60 | 62.86 | 75.71 | **80.72** |
| MLL | DT | 66.67 | 93.33 | 80 | 66.67 | 66.67 | 60 | 86.67 | 73.33 | **93.33** | **93.33** |
|  | SVM | 66.67 | 80 | 93.33 | 80 | 80 | 86.67 | 86.67 | 86.67 | 93.33 | **95.55** |
|  | LR | 80 | 86.67 | 66.67 | **93.33** | 86.67 | 80 | 80 | 86.67 | **93.33** | **93.33** |
| Ovarian | DT | 100 | 96.08 | 94.12 | 96.08 | 98.04 | 96.08 | 94.12 | 96.08 | **100** | **100** |
|  | SVM | **100** | **100** | **100** | **100** | **100** | **100** | **100** | 98.04 | **100** | **100** |
|  | LR | **100** | **100** | 98.04 | 94.12 | **100** | 84.31 | **100** | **100** | **100** | **100** |
| Leuk | DT | 73.33 | 93.33 | 93.33 | 93.33 | 73.33 | 73.33 | 93.33 | 93.33 | 96.66 | **96.66** |
|  | SVM | 93.33 | 86.67 | 86.67 | 80 | 86.67 | 86.67 | **100** | **100** | **100** | **100** |
|  | LR | 60 | 86.67 | **100** | 93.33 | 86.67 | **100** | 93.33 | **100** | **100** | **100** |
| Winner | DT | 0 | 0 | 0 | 0 | 0 | 0 | 0 | 0 | 2 | 7 |
|  | SVM | 1 | 1 | 1 | 1 | 1 | 1 | 2 | 1 | 2 | 7 |
|  | LR | 1 | 1 | 1 | 1 | 1 | 1 | 1 | 2 | 3 | 7 |
